# Lower Limb Kinematics Using Inertial Sensors during Locomotion: Accuracy and Reproducibility of Joint Angle Calculations with Different Sensor-to-Segment Calibrations

**DOI:** 10.3390/s20030715

**Published:** 2020-01-28

**Authors:** Julien Lebleu, Thierry Gosseye, Christine Detrembleur, Philippe Mahaudens, Olivier Cartiaux, Massimo Penta

**Affiliations:** 1 Neuro Musculo Skeletal Lab (NMSK), Institut de Recherche Expérimentale et Clinique, Université Catholique de Louvain, Secteur des Sciences de la Santé, Avenue Mounier 53, B-1200 Brussels, Belgium; christine.detrembleur@uclouvain.be (C.D.); philippe.mahaudens@uclouvain.be (P.M.); crt@ecam.be (O.C.); 2 Institue of Neurosciences (IONS), Université Catholique de Louvain, Secteur des Sciences de la Santé, Place Pierre de Coubertin 1, B-1348 Louvain-la-Neuve, Belgium; thierry@arsalis.com (T.G.); massimo@arsalis.com (M.P.); 3 Arsalis SPRL, Chemin du Moulin Delay 6, B-1473 Glabais, Belgium; 4 Cliniques Universitaires St-Luc, Service D’orthopédie, avenue Hippocrate 10, B-1200 Brussels, Belgium; 5 ECAM, Brussels Engineering School, Promenade de l’Alma 50, B-1200 Brussels, Belgium

**Keywords:** inertial sensor, gait, validity, functional calibration, accuracy, wearable electronic devices

## Abstract

Inertial measurement unit (IMU) records of human movement can be converted into joint angles using a sensor-to-segment calibration, also called functional calibration. This study aims to compare the accuracy and reproducibility of four functional calibration procedures for the 3D tracking of the lower limb joint angles of young healthy individuals in gait. Three methods based on segment rotations and one on segment accelerations were used to compare IMU records with an optical system for their accuracy and reproducibility. The squat functional calibration movement, offering a low range of motion of the shank, provided the least accurate measurements. A comparable accuracy was obtained in other methods with a root mean square error below 3.6° and an absolute difference in amplitude below 3.4°. The reproducibility was excellent in the sagittal plane (intra-class correlation coefficient (ICC) > 0.91, standard error of measurement (SEM) < 1.1°), good to excellent in the transverse plane (ICC > 0.87, SEM < 1.1°), and good in the frontal plane (ICC > 0.63, SEM < 1.2°). The better accuracy for proximal joints in calibration movements using segment rotations was traded to distal joints in calibration movements using segment accelerations. These results encourage further applications of IMU systems in unconstrained rehabilitative contexts.

## 1. Introduction

Quantitative assessment of lower limb kinematics is required in various applications, such as motion analysis, sports science, and rehabilitation. Although opto-electronic motion capture systems are considered the gold standard for this assessment, their widespread use is limited by their restricted area of measurement, their optical limitations due to marker occlusion or reflection, and their cost. Moreover, opto-electronic trackers are generally used in a restricted lab environment, which further limits the exploration of real-life movements and exercises. Wearable sensors, such as inertial measurement units (IMUs), have been developed to overcome these limitations [1], allowing for human motion analysis in unconstrained real-life conditions [2,3].

Although they generally contain a 3D accelerometer, a 3D gyroscope, and an optional 3D magnetometer, an IMU does not measure joint angles perfectly. Joint angles obtained via signal integration typically drift over time [4,5] and their accuracy varies with the joint assessed and the movement complexity [6,7]. While an error under 5° is generally accepted for most clinical gait applications [8], the measurement error typically ranges from 5° to 18.8° depending on the joint and the plane of motion [6]. Another major challenge in IMU-based human motion analysis that the IMUs’ local coordinate systems are not aligned with physiologically meaningful axes [9]. Such alignment, required to compute joint angles, can be performed via a “sensor-to-segment” calibration procedure [10].

The first approach to ensure this alignment consists of a rigorous positioning of the sensor in relation to the anatomy [11]. This method assumes that the segment axes are parallel to the IMU axes, is approximate, and requires user expertise to locate the sensor axes relative to the joint axes for both segments around each joint. The second approach consists of placing an IMU on each segment and aligning the IMU and joint axes via a set of calibration postures [12] and/or movements around physiological motion axes. The latter functional method [13,14] consists of making the subject stand upright with straight legs for a few seconds to define the vertical axis for each IMU or segment, while the other axes are defined via active or passive movements [13,14]. Since the movements are generally human-controlled, the accuracy of the axes definition essentially relies on the subjects’ ability to precisely hold a given posture and on the execution of a given movement [9]. The third approach consists of exploiting the kinematic constraints of the joints and use almost arbitrary movements to perform the sensor-to-segment calibration [9,15]. This method is particularly adapted to single axis joints that can be satisfactorily modelled as a hinge joint like the knee; however, the modeling of spherical joints requires the execution of movements mostly around one axis to identify the joint axes [9], which resemble the functional method.

The IMU-based tracking of lower limb spherical joints, using one of the two aforementioned methods, therefore requires the execution of a functional calibration movement. While the accuracy of the sensor-to-segment calibration is determined by the quality of the functional calibration movement, to our knowledge, it has only been investigated in one study for upper limb motion tracking [16] and no study has compared functional calibration movements for the tracking of lower limb joint angles.

This study aimed to (1) assess the accuracy of different functional calibration methods in order to compute the lower limb joint angles during walking, (2) assess the reproducibility of different functional calibration movements, and (3) compare the accuracy provided by functional calibration movements in different gait movements.

## 2. Materials and Methods

### 2.1. Participants

Seven healthy young adults participated in this study (6 females, 1 male, mean (SD) age = 22.6 (1.5) years, height = 1.67 (0.08) m, body mass = 65.4 (11.6) kg). Participants were included in the study if they were between 20 and 25 years old and free of any injury at the time of participation. The study protocol was approved by the ethics committee of our university (agreement number: B403201523492) and each patient provided written informed consent to the use of their anonymized data.

### 2.2. Experimental Setup and Recordings

**Figure 1:**
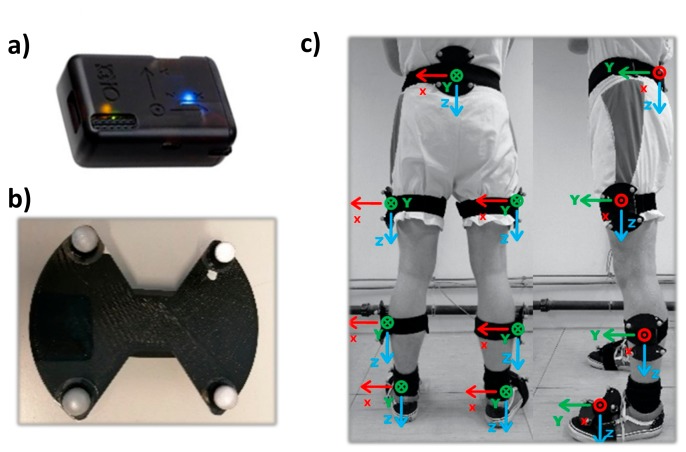
Sensor locations: (**a**) x-IMU from xi-o Technologies, (**b**) 3D-printed **a**crylonitrile **b**utadiene **s**tyrene (ABS) enclosures (4 markers of 14 mm diameter) with the inertial measurement unit (IMU) reference frame, and (**c**) segment reference frame of the 7 IMUs on the subject.

To assess the lower limb joint kinematics, seven wearable IMUs; (x-IMU, x-io Techologies, Bristol, UK) were fixed in matched 3D-printed ABS (acrylonitrile butadiene styrene) enclosures and attached by means of a semi-elastic belt to seven lower body segments, as shown in Figure 1: the waistline at the level of the fifth lumbar vertebra (L5), the middle of the thighs, the middle of the shanks, and at the dorsal side of the feet. The IMUs were firmly strapped on the skin or clothes. Although this could lead to undesirable artifacts, it is more representative of records in an unconstrained context, such as outdoor conditions. Each IMU included a tri-axial accelerometer (full scale ±6 g), a gyroscope (±2000°/s), and a magnetometer (±8.1 G) that were sampled at a frequency of 128 Hz. The IMUs were connected to a computer by means of a Bluetooth connection using a custom application based on open source software [17] (C# program, github.com/xioTechnologies). Each movement was recorded independently. The synchronization between the IMUs was ensured by a custom-built magnetic coil that sent a magnetic impulse at the beginning of each recording.

Four reflective markers were fixed at each corner of the ABS enclosures to define clusters for each segment (Figure 1). Motion capture data were collected at a rate of 200 Hz using an eight-camera motion analysis system (Vicon V5 Motion Systems, Oxford Metrics Ltd., Oxford, UK)) and processed using Nexus 2.5 software. The position of each marker on the cluster allowed for the orientation of each segment to be computed in the lab reference frame.

### 2.3. Functional Calibration and Test Movements

**Figure 2:**
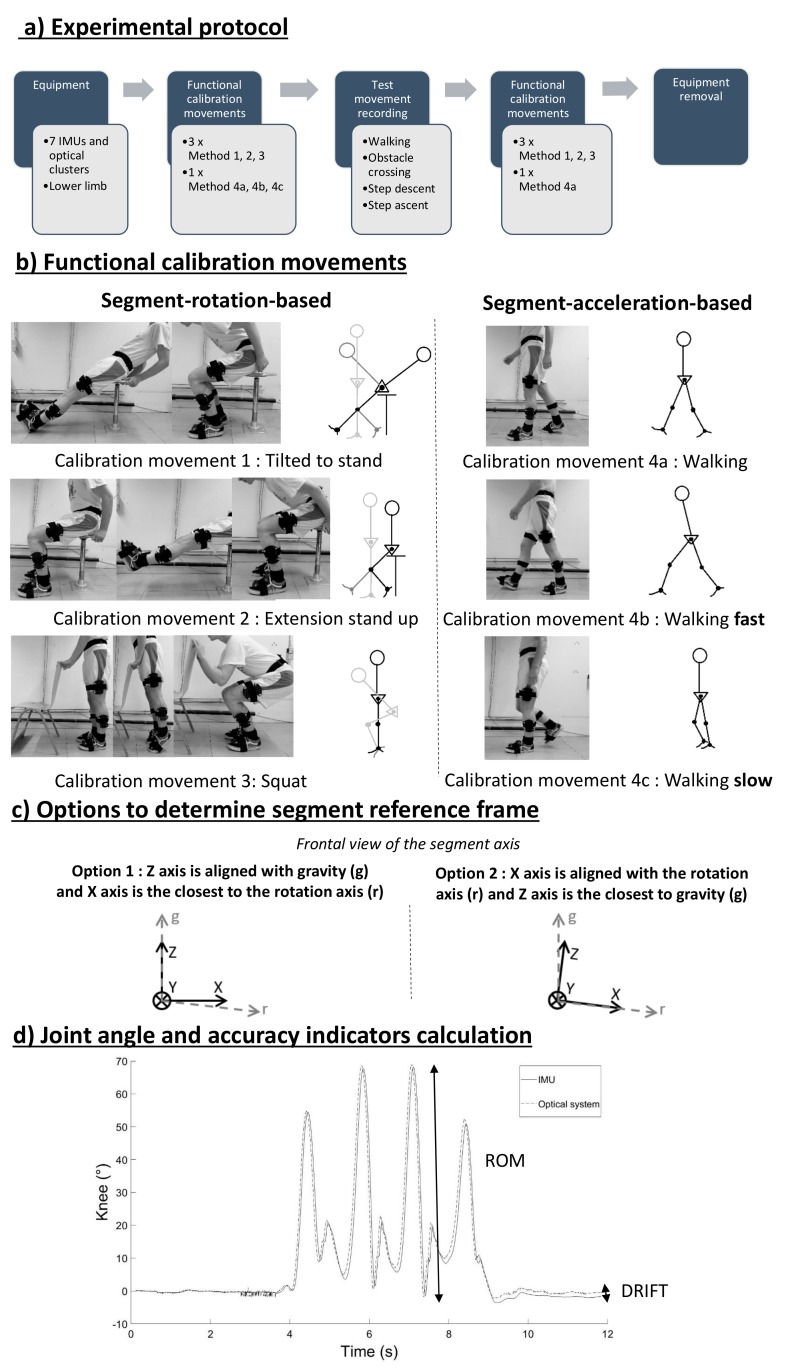
(**a**) Experimental protocol. (**b**) Functional calibration movements: for movements 1, 2, and 3, where the second axis was defined as the principal rotational axis as determined by a principal component analysis (PCA) on gyroscope signals; for movements 4a, 4b, and 4c, the second axis was defined as the principal acceleration axis through a PCA on accelerometer signals. (**c**) Two options to determine the segment reference frames, as shown in segment frontal views. (**d**) The accuracy was assessed using the root mean square error (RMSE), the absolute difference in the range of motion (ΔROM) between both systems, and the absolute drift accumulated during the movement (DRIFT).

The experimental protocol is illustrated in Figure 2. Four functional calibration movements were performed to assess their reproducibility and accuracy regarding lower limb joint angle measurement with the IMUs. These movements were designed to include a rotation in the sagittal plane of each lower body segment, including the pelvis, while being easy to explain and reproduce. Each functional calibration movement included (1) an upright static posture with the arms alongside the body and the feet parallel beside each other that was used to define the segment vertical axis and (2) a functional movement spanning a range of orientations for each segment in the sagittal plane that was used to define a second segment axis. In the static posture, the segment was supposed to have a zero angle in all three planes such that the segment reference frames were aligned with the lab reference frame. The X axis was defined as the medio-lateral axis, pointing to the left of the subject; the Y axis as the anterio-posterior axis, pointing in front of the subject; and the Z axis as the vertical axis, pointing downward (Figure 1c).

The instructions were as follows:

- Calibration movement 1: “Tilted to stand”: Start in a leaned-back position with extended legs on the chair, bend the knees, bend the trunk forward, get up from the chair, and stop moving.
- Calibration movement 2: “Extension stand up”: Sit on the chair, extend the knees in front of you, bend the knees, bend the trunk forward, get up from the chair, and stop moving.
- Calibration movement 3: “Squat”: Stand in front of the chair, rise on your heels, squat deeply, get up, and stop moving.
- Calibration movement 4: “Walking” 5 m:
- 4a. Walk at your pace to the red line on the floor, then stop moving.
- 4b. Fast: walk five meters as fast as you can without running to the red line on the floor as if you were late to a meeting.
- 4c. Slow: walk five meters slowly to the red line on the floor but keep moving.

The functional calibration movements were demonstrated by the operator and each participant received practice trials to get used to each movement. Each functional calibration movement was recorded three times before and three times after the execution of the test movements. The walking movement at self-selected speed was only performed two times, before and after the test movements.

Four test movements were performed in the same order:

- walking five meters at a self-selected speed;
- stepping over an obstacle 28 cm in height while walking 5 m at a self-selected speed;
- ascend a step 20 cm in height;
- descend a step 20 cm in height.

The mean recorded times for test movements were 11 s for walking, 11 s for stepping over an obstacle, 14 s for the step ascent, 16 s for the step descent.

### 2.4. Signal Processing

An open source attitude and heading reference system (AHRS) algorithm was used for sensor fusion between the accelerometer and gyroscope sensor data of the IMU (Mahony’s AHRS algorithm) [18]; the magnetometer signals were omitted. The four calibration movements were used to compute the orientation of each segment relative to the lab reference frame in different ways. The gravity vector during the static upright posture was used to define the vertical axis for each segment. A second segment axis was defined in one of two ways depending on the method of functional calibration. For functional calibration movements 1, 2, and 3, it was defined as the principal rotational axis as determined by a principal component analysis (PCA) on gyroscope signals. Two options were used to determine the segment reference frame (see frontal views of the segment reference frame in Figure 2c): either the gravity vector (g) was defined as the vertical axis and the lateral axis was forced to be the orthogonal axis closest to the rotation axis of the functional calibration movement (r), or the lateral axis was defined as the functional calibration movement rotation axis and the vertical axis was the orthogonal axis closest to the gravity vector (and thus the transversal plane was not perfectly horizontal in the static upright posture). For functional calibration movements 4a, 4b, and 4c, the second axis was defined as the principal acceleration axis through a PCA on accelerometer signals transformed in a lab-fixed reference frame. The 3D orientation of the pelvis and joint angles for the hips, knees, and ankles were calculated from the segment orientations based on the recommendations of the International Society of Biomechanics [6] for the different functional calibration movements. Flexion-extension were rotations around the X axis, abduction-adduction was around the Y axis, and internal-external rotations were around the Z axis.

The lower body 3D kinematics derived from the optical system were computed in two different ways. They were either computed in the lab frame or computed through the same functional calibration procedures described above, using the principal axis of rotation or acceleration determined from optical records.

For each participant, a static period (about 5 s) in a standing position was captured at the beginning of each test to define the segment’s initial orientation for the IMU AHRS algorithm.

### 2.5. Data Analysis

Joint angles of the walking test movement were calculated for all functional calibration procedures. The accuracy of the IMU kinematics was computed for each calibration procedure as the difference in joint angle between the IMU and optical measurements. The accuracy was assessed using the root mean square error (RMSE) during the movement period, the absolute difference in the range of motion (ROM) between both systems (ΔROM), and the absolute drift accumulated during the movement due to the error in the angular rate integration (DRIFT). The RMSE, ΔROM, and DRIFT parameters were computed using Matlab 2018 (Mathworks Inc, Natick, MA, USA) and are expressed in degrees.

A generalized linear model was used to assess the effect of (1) the functional calibration movement, (2) the option used to determine the segment’s reference frame to compute the IMU orientation, and (3) the functional calibration method for the optical system on the amplitude of the RMSE, ΔROM, and DRIFT parameters for each joint angle and plane of motion. This analysis was performed with SPSS (version 25, IMB Corporation, Amonk, NY, USA) and the significance level was set to α = 0.05.

The reproducibility of each functional calibration movement was assessed as the difference in joint angle computed from each repetition of the functional calibration movement. The reproducibility of the ROM parameter in all movement planes and joints was determined based on the intra-class correlation coefficient (ICC) [19] and standard error of measurement (SEM) [19]. Values of ICC ≥ 0.90 were considered as excellent, 0.70–0.89 as good, 0.40–0.69 as acceptable, and <0.40 as low [20]. The SEM estimates the non-systematic variance and reflects the within-subject variability among repeated calibrations. A proportional SEM (SEM%) was calculated by expressing the SEM relative to the mean ROM (SEM% = (SEM/mean) × 100%)) [21]. An SEM% above 10% was considered as high.

Once the most accurate and reproducible functional calibration method was selected for a walking test, the accuracy was determined for the other test movements, namely the step ascent, descent, and stepping over an obstacle, using the RMSE and ΔROM parameters. The parameters were calculated for the front leg (i.e., the first leg to touch the step in the step ascent, the first leg to touch the floor leg in the step descent, and the first leg to touch the ground in obstacle stepping) and for the back leg in the different test movements. Differences in the RMSE and ΔROM parameters between test movements were assessed with a one-way ANOVA. Tukey’s post hoc test was used to reveal which groups differed in the case of significant *p*-values. The significance level was set to α = 0.05.

## 3. Results

**Figure 3:**
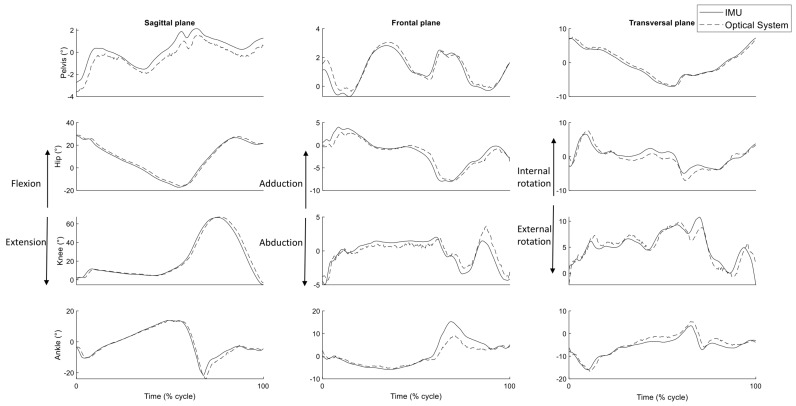
Typical trace of the 3D pelvis orientation and joint angles during a gait stride for measurements with the IMUs (plain lines) and with the optical system (short dash lines) using the walking functional calibration.

The pelvis orientation and the joint angles during a time-normalized typical gait stride at a self-selected speed of 1 m·s^−1^ are illustrated in Figure 3 for measurements with the IMUs and with the optical system using the walking functional calibration (Calibration movement 4a). The traces displayed a classical movement pattern as indicated by the similarity between both set of measurements, with a mean RMSE value lower than 5° for all joints in all planes.

### 3.1. Assessment of the IMU Accuracy

**Figure 4:**
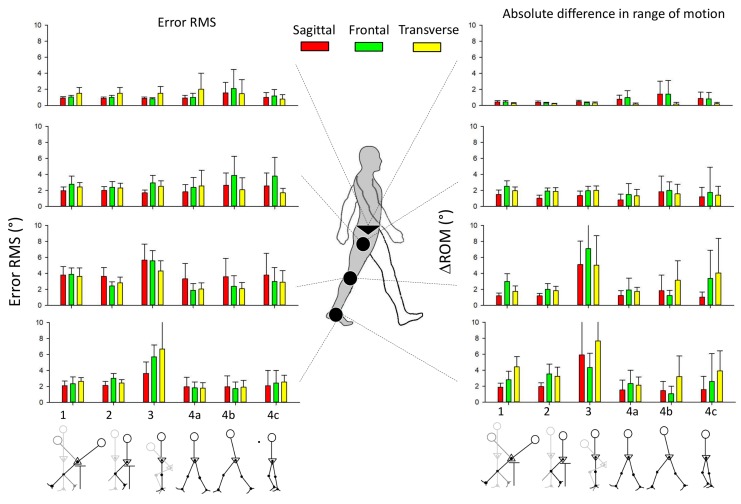
Accuracy across functional calibration movements (1. Tilted to stand, 2. Extension, 3. Squat, 4a. Walking (1 ± 0.1 m·s−1), 4b. Walking fast (1.5 ± 0.1 m·s−1), 4c. Walking slow (0.6 ± 0.1 m·s−1)). Error bars are the confidence interval means at 95%.

The mean differences between measurements from the IMUs and from the optical system, as well as the factors affecting this difference, are presented in Table 1. The highest mean error was observed at the knees and at the ankles, bilaterally, as shown by the RMSE between 3.0° and 4.1° and by the ΔROM between 1.9° and 5.1° in any plane of motion. The generalized linear model also indicated that these differences were significantly linked to the functional calibration movement; see the details in Figure 4. These errors were also significantly associated with the methods of determination of the IMUs reference frame, although to a lesser extent: the mean (SD) RMSE was 2.3° (1.7°) when the vertical axis was aligned to the gravity vector and 3.0° (3.1°) when the lateral axis was aligned to the segment rotation axis. The errors were not significantly linked to the two different methods used to compute the 3D kinematics from the optical system (lab frame or computed through the functional calibration). Table 1 also shows that the DRIFT was largely independent of the factors considered in the generalized linear model. 

The variation of the RMSE across different functional calibration movements is illustrated in Figure 4. While the RMSE varied between approximately 1° and 6° for most joints, planes of motion, and functional calibration movements, it was larger for the “squat” calibration movement, especially at the knee and ankle where the upper confidence limit of the RMSE reached up to 8° and the upper confidence limit ΔROM reached up to 10° or more. Tilted, extension, and walking functional calibration movements tended to provide less accurate measurements at the knee. Walking functional calibration movements tended to report more accurate angles for distal lower limb joints, although without a clear visible impact on the ROM accuracy. After excluding the “squat” movement, the mean accuracy for all other calibration movements is summarized in Table 2, indicating a mean RMSE of less than 2° at the pelvis, less than 3° at the hip and ankle, and less than 4° at the knee with a trend for larger errors in the frontal plane. The mean errors were smaller in ΔROM. DRIFT values at the hips and at other lower limb joints in the sagittal plane were on average under 2.7°, while higher mean values up to 4.9° were observed at the knee and at the ankle in the frontal and transverse planes.

### 3.2. Assessment of the IMU Reproducibility

The mean ROM recorded for each joint in each plane of motion during one walking test movement are presented in Table 3, together with the reproducibility indices computed across repetitions of each calibration movement. The mean ROM displayed symmetrical values for both limbs and classical movement amplitudes for walking at 1 m·s^−1^. Overall, the mean reproducibility was excellent for all calibration movements in the sagittal plane (ICC: 0.96–0.99) and it was good to excellent in the transverse plane (ICC: 0.87–0.93). In the frontal plane, the mean reproducibility was good for all calibration movements (ICC: 0.79–0.86), except for walking, which had an acceptable reproducibility on average (ICC: 0.63) due to the low ICC observed for the hip and knee. The reproducibility was uniformly good to excellent across the calibration movements, except for the walking movement, which reported slightly lower reproducible movement amplitudes for proximal joints (as low as ICC = 0.76 for the hip compared to ICC = 0.94 for any other movement) and slightly higher reproducibility in distal joints (as high as ICC = 0.91 for the ankle compared to ICC = 0.82 for other movements). For all functional calibration movements, whatever the joint, the mean SEM was within 1.2° in all planes, although the mean SEM remained generally higher at the knee (0.9°) and ankle (1.8°) compared to the hip (0.6°) and pelvis (0.1°). This resulted in acceptable variations between movements, as shown by a mean SEM% of 1.8% in the sagittal plane, 4.8% in the transverse plane, and 7.9% in the frontal plane, where articular amplitudes were smaller for all lower limb joints.

### 3.3. Assessment of Accuracy in Different Test Movements

The functional calibration movements that provided the highest accuracy were the tilted and extension movements, as well as walking at a self-selected speed (mean RMSE for all joints, respectively: 2.5°, 2.3°, 2.2°). Since these calibration movements provided a comparable performance for the functional calibration of the IMU, the mean accuracy with the walking functional calibration was computed for the measurement of the four gait test movements, namely walking, ascending or descending one step, and stepping over an obstacle, when considering the lateral axis of the reference frame perpendicular to the gravity vector (as similar accuracies were obtained when considering it parallel to the axis of the segment rotation during the functional calibration). The mean accuracy obtained with the functional calibration movements retained is presented in Table 4. The RMSE and ∆ROM were both smaller than 6° for ascending a step and smaller than 13° for descending it. For stepping over an obstacle, the RMSE reached 13° but the ∆ROM had maximum values of only 4°. Notably, the accuracy of the IMU measurements were higher in walking than in other gait movements, where larger inaccuracies were observed, especially for a step ascent (both ankles in the sagittal plane and back leg hip in the frontal plane), for a step descent (all joints of both legs), and for stepping over an obstacle (all joints of both legs excluding the pelvis), although the error was generally within 5° and only rarely exceeded 10°.

## 4. Discussion

The main objective of this study was to compare the accuracy and reproducibility of lower limb joint angles computed from IMUs following different functional calibration methods. The study showed that applying a functional calibration movement before IMU-based lower limb kinematic assessment allowed for a fairly accurate measurement of gait movements. Except for the squat calibration movement, only small discrepancies were observed between functional calibration movements during a walking task, with a peak mean error of 3.6° for any joint in any plane of movement. Overall, the absolute reproducibility was similar for the three planes, but relative reproducibility was higher in the sagittal plane, with a mean standard error of measurement of less than 1.1° observed between multiple repetitions of the same functional calibration movement. A comparable overall performance was observed for different calibration movements, although each movement reported variable merits for different joints and planes of movement. Although the highest accuracy was observed in straight walking with a mean error of 2.2°, more complex gait movements tended to provide larger but limited errors, with a mean error of 3.5° for a step ascent, 5.4° for a step descent, and 4.7° for crossing an obstacle.

### 4.1. Accuracy of Different Calibration Methods during Straight Walking

The accuracy reported in this study during straight walking ranged from 1.1° to 3.6° for RMSE and from 0.2° to 3.4° for ∆ROM, which is comparable to the mean error below 3° reported when using marker clusters on segments [22] rather than markers on anatomical landmarks [23,24,25]. Indeed, both methods reported different joint kinematics and accounted differently for errors of markers placement, soft tissue artefacts, and biomechanical model calculations [22]. The functional calibration of the optical system did not influence the accuracy, indicating that the reference frame obtained for each segment with the functional calibration movements were close to the optical reference frame. As the magnetometer was not used in the AHRS algorithm, the DRIFT was controlled to be acceptable (mean of 2.3°) for such short experiments. The drift was slightly higher in the more distal joints, probably due to the higher speed of the movements [26,27]. The drift was slightly lower in the sagittal plane, probably because the drift in this plane was better compensated by the sensor fusion algorithm. Although a mean error under 2° has been obtained on a single-joint movement [13], the accuracy obtained with our multi-joint model is acceptable for most clinical gait applications [8].

While the tilted and extension calibration movements provided a higher accuracy in the hip and ankle kinematics compared to the knee, walking calibration movements reported a higher accuracy for distal joints, whatever the walking speed. This observation can be supported by (1) a greater variability in the knee kinematics during the tilted and extension movements compared to straight walking and (2) higher accelerations of the distal relative to the proximal segments during walking. This observation also showed that the reference frame for each segment can be equally determined via a rotational movement recorded by the gyroscopes or via a translational movement recorded by the accelerometers contained in each IMU. The accuracy obtained in slow walking also validates the use of this functional calibration movement in similar conditions, which is often encountered in pathological gaits or in older adults [28].

The lower accuracy reported for the knee and ankle via the squat calibration movement could be explained by the lower movement amplitude of the shank and foot segments during this calibration movement. Indeed, the smaller amplitude-to-noise ratio probably resulted in an erroneous definition of the reference frame, leading to kinematic crosstalk [29]. This result also showed that functional movements exploring a wide range of segment orientation tended to provide more accurate segment reference frames.

### 4.2. Reproducibility of Calibration Movements

Reproducibility was excellent in 65% of the tested joints and motion planes, good in 24%, acceptable in 10%, and poor in 1 observation out of 84. Concerning differences between calibration movements, the walking calibration movement produced the highest reproducibility and SEM% for the ankle, while the other functional calibration movements produced higher reproducibility indices for the pelvis and hip joints. The lower reproducibility at the ankle for the segment-rotation-based movements could be explained by the difficulty in reproducing movements purely in the sagittal plane. This observation also supports previous results showing that the variable position of the foot affects the functional calibration when using different static postures [12]. The use of more guidance or more repetitions of the calibration movements could improve the reproducibility by (1) avoiding parasitic movements of the feet out of the sagittal plane and (2) decreasing the impact of any parasitic movement on the definition of the rotation axis. However, in order to limit the complexity and burden of the functional calibration movements, the walking calibration movement remains a remarkably convenient alternative since it offers a good to excellent reproducibility (though lower than other movements for the proximal joints), with a very simple and ecological movement. Caution may be needed for subjects having an impaired walking pattern, e.g., a subject walking with the feet pointing outwards.

A higher reproducibility was observed in the sagittal plane compared to the frontal plane. This could be explained by the higher range of motion in the sagittal plane during walking, leading to more kinematic crosstalk in the other planes measured and/or by the fact that the functional calibrations mainly generated segment movements in the sagittal plane. The combination of the higher variability and smaller ROM in the frontal plane during walking led to a higher SEM% in this plane, as also shown in upper limb anteroposterior reaching tasks [19]. Higher SEM% values inevitably require higher changes to detect meaningful functional changes, e.g., after therapy. The reproducibility of calibration movements in the frontal plane should be explored for the assessment of functional outcomes involving larger movements in the frontal plane.

### 4.3. Accuracy across Different Gait Movements

More complex gait movements tended to provide larger errors than a peak mean RMSE of 3.6° and a peak mean ∆ROM of 3.4° for straight walking. Indeed, the peak mean errors obtained in the sagittal plane for a step ascent of 3°, 5°, and 5° for hip, knee, and ankle, respectively, correspond to errors in elevation angles of 5°, 4°, and 4° previously reported for the same joints [30]. Similarly, the peak mean ∆ROM of 6.4° obtained for the stair ascent and of 4.6° for the stair descent are comparable to the errors previously reported for healthy subjects (peak error of 4.1° for a stair ascent and 4.8° for a stair descent) [31]. Therefore, before implementing inertial sensors in a complex, real-life context, the accuracy should be established in such a context rather than extrapolated from simpler gait movements recorded in controlled lab conditions.

### 4.4. Limitations and Perspectives

This study focused on healthy adults and this could be a limitation in case the functional calibration movements proposed here would be used with patients with a limited range of motion or who have parasitic movements that may hinder an accurate and reproducible calibration movement. The transferability to the elderly or to patients with motion disabilities should be assessed in further studies.

The IMU magnetometer was voluntarily omitted in this study in order to avoid ferromagnetic disturbances. The recordings in this study were limited in time due to the short time required to execute the investigated movements. The drift resulting from longer records [32] could be limited by using the IMU magnetometer or algorithms that constantly fuse the segment’s angular velocity and linear acceleration via known kinematic relations between segments [33]. 

Although a high accuracy for the lower limb joint angles has been obtained by using only the gyroscope signals, our methods could be improved by also accounting for the segment accelerations [9,34], which can be used to locate the joint centers and improve the robustness of the segment orientations [35]. Another approach consists in using a hinge joint model and kinematic constraints to develop automatic or so-called “plug and play” calibrations [9,36]. This less restrictive method may facilitate clinical applications where patients with motion disabilities cannot be expected to perform precise prescribed calibration movements.

## 5. Conclusions

This study documents the high accuracy of IMU-derived lower limb joint angles during walking using several functional movements for the sensor-to-segment calibration. Functional movements requiring larger segmental angular amplitudes provided more accurate segmental reference frames and led to a higher accuracy regarding the kinematics of the adjacent joints. Alternatively, the higher linear accelerations generated at distal segments during a walking functional calibration also led to a higher reproducibility for distal joints, in comparison to the functional calibration based on the principal rotational axes. The walking, tilted, and extension functional calibration movements were shown to be three equivalent options for the gait movement examined in this study. In addition, for examining walking, the walking functional calibration may be superior because it involved very limited material and instruction complexity, which strengthens its interest in uncontrolled environments.

## Figures and Tables

**Table 1:** Generalized linear model: effect of functional calibration movement, segment reference frame, and optical functional calibration on the accuracy.

Accuracy Indicator		RMSE (°)				∆ROM (°)				DRIFT (°)			
Calibration Factor			Functional Calibration Movement	Segment Reference Frame	Optical Functional Calibration		Functional Calibration Movement	Segment Reference Frame	Optical Functional Calibration		Functional Calibration Movement	Segment Reference Frame	Optical Functional Calibration
		Mean (SD)	Mean Difference between Methods			Mean (SD)	Mean Difference between Methods			Mean (SD)	Mean Difference between Methods		
Pelvis	Sagittal	0.9 (0.5)	-	-	-	0.4 (0.6)	1.0	-	-	0.4 (0.6)	-	-	-
	Frontal	1.1 (0.9)	1.3	-	-	0.5 (0.7)	1.1	-	-	0.5 (0.7)	-	-	-
	Transverse	1.5 (1.8)	-	-	-	0.2 (0.2)	-	-	-	0.2 (0.2)	-	-	-
Hip right	Sagittal	2.0 (1.2)	-	-	-	1.6 (1.6)	-	1.0	-	1.6 (1.6)	-	-	-
	Frontal	2.7 (2.1)	-	-	-	2.3 (1.5)	-	-	-	2.3 (1.5)	-	-	-
	Transverse	2.4 (1.5)	-	-	-	2.2 (1.3)	-	-	-	2.2 (1.3)	-	-	-
Knee right	Sagittal	**4.1 (3.1)**	-	1.5	-	2.4 (3.8)	**3.9**	-	-	2.4 (3.8)	1.2	-	-
	Frontal	**3.6 (2.3)**	**4.0**	-	-	**4.7 (6.4)**	**9.2**	-	-	**4.7 (6.4)**	-	-	-
	Transverse	**3.3 (2.1)**	-	2.4	-	**3.2 (5.2)**	**4.3**	**3.1**	-	**3.2 (5.2)**	-	-	-
Ankle right	Sagittal	2.5 (1.7)	-	0.9	-	2.7 (5.1)	-	-	-	2.7 (5.1)	-	-	-
	Frontal	**3.3 (2.5)**	**3.7**		-	**4.2 (5.6)**	**7.2**	-	-	**4.2 (5.6)**	-	-	0.5
	Transverse	**2.4 (4.3)**	**4.3**	2.6	-	**5.1 (6.6)**	**5.6**	2.8	-	**5.1 (6.6)**	2.0	1.0	-
Hip left	Sagittal	2.5 (0.8)	-	-	-	0.8 (0.7)	-	-	-	0.8 (0.7)	-	-	-
	Frontal	2.1 (1.6)	-	-	-	1.9 (1.7)	-	-	-	1.9 (1.7)	-	-	-
	Transverse	2.1 (1.6)	-	-	-	2.0 (1.8)	1.9	0.6	-	2.0 (1.8)	-	-	-
Knee left	Sagittal	**3.7 (3.5)**	-	1.9	-	1.9 (3.6)	-	-	-	1.9 (3.6)	-	-	-
	Frontal	**3.5 (1.7)**	1.9	-	-	**4.3 (4.0)**	**4.9**	-	-	**4.3 (4.0)**	-	-	-
	Transverse	**3.0 (1.6)**	-	1.4	-	2.7 (3.9)	3.0	2.6	-	2.7 (4.0)	-	-	-
Ankle left	Sagittal	**3.2 (1.5)**	-	-	-	2.3 (4.0)	**4.6**	2.2	-	2.3 (4.0)	-	-	-
	Frontal	2.9 (1.6)	2.8	-	-	**3.6 (3.9)**	**3.8**	-	-	**3.6 (3.9)**	-	-	-
	Transverse	**3.2 (4.3)**	**4.8**	2.4	-	**3.6 (5.1)**	**6.7**	2.0	-	**3.6 (5.1)**	-	-	-

Numerical values indicate significant effects, dashes indicate non-significant effects. Mean differences between calibration factors are presented in degrees for significant effects (*p* < 0.05) and in bold for angles >3°. For the functional calibration movement factor, the highest mean difference between the six movements is presented. RMSE: root mean square error, ∆ROM: absolute difference in range of motion, DRIFT: absolute difference between IMU and optical system at end of recording, SD: standard deviation.

**Table 2:** Summary of the accuracy without the squat calibration movement and with reference frames aligned with gravity.

		RMSE (°)	∆ROM (°)	DRIFT (°)
		Mean (SD)		
Pelvis	Sagittal	1.0 (0.7)	0.7 (0.8)	0.4 (0.6)
	Frontal	1.2 (1.1)	0.7 (0.9)	0.5 (0.4)
	Transverse	1.5 (1.8)	0.2 (0.2)	0.2 (0.2)
Hip right	Sagittal	2.1 (1.3)	1.0 (1.0)	1.6 (1.6)
	Frontal	2.9 (2.2)	2.0 (1.7)	2.3 (1.6)
	Transverse	2.2 (1.5)	1.7 (1.1)	2.1 (1.3)
Knee right	Sagittal	**3.6 (2.5)**	1.1 (0.6)	1.9 (1.8)
	Frontal	2.8 (1.6)	2.5 (2.3)	**4.0 (5.3)**
	Transverse	2.2 (1.2)	1.8 (1.9)	2.9 (4.8)
Ankle right	Sagittal	2.0 (1.3)	1.6 (1.1)	2.7 (5.4)
	Frontal	2.4 (1.6)	2.6 (2.7)	**3.5 (4.6)**
	Transverse	2.2 (1.0)	**3.4 (2.9)**	**4.9 (6.6)**
Hip left	Sagittal	2.5 (1.7)	1.1 (0.7)	0.8 (0.7)
	Frontal	2.4 (1.9)	1.8 (1.8)	2.0 (1.7)
	Transverse	2.0 (1.5)	1.5 (1.3)	2.0 (1.8)
Knee left	Sagittal	**3.2 (2.6)**	1.0 (0.7)	1.4 (1.1)
	Frontal	**3.2 (1.7)**	**3.1 (3.0)**	**4.0 (3.9)**
	Transverse	2.3 (0.9)	1.5 (1.1)	2.5 (3.9)
Ankle left	Sagittal	2.9 (1.4)	0.9 (0.8)	2.1 (4.1)
	Frontal	2.3 (1.2)	2.3 (1.9)	**3.1 (3.3)**
	Transverse	1.9 (0.8)	1.7 (1.7)	**3.1 (5.1)**

Bold values indicate angles >3°.

**Table 3:** Joint angle measurement reproducibility for one walk task across four functional calibration movements.

		ROM (°)		Tilted (Movement 1)			Extension (Movement 2)			Squat (Movement 3)			Walking (Movement 4a)	
		Mean (SD)	ICC	SEM (°)	SEM%	ICC	SEM (°)	SEM%	ICC	SEM (°)	SEM%	ICC	SEM (°)	SEM%
Pelvis	Sagittal	*12.8 (2.1)*	1.00	0.1	0.7%	1.00	0.1	0.5%	1.00	0.1	0.5%	0.98	0.3	2.5%
	Frontal	*6.8 (0.8)*	0.98	0.1	1.7%	0.98	0.1	1.6%	1.00	0.1	0.8%	0.76	0.4	6.1%
	Transverse	*17.9 (4.1)*	1.00	0.0	0.0%	1.00	0.0	0.0%	1.00	0.0	0.0%	1.00	0.0	0.0%
Hip right	Sagittal	*50.4 (2.4)*	0.98	0.3	0.7%	1.00	0.2	0.4%	1.00	0.3	0.6%	0.90	0.9	1.8%
	Frontal	*14.0 (1.9)*	0.81	0.8	5.8%	0.90	0.7	5.1%	0.90	0.6	4.2%	0.50	1.3	9.3%
	Transverse	*18.4 (2.7)*	0.98	0.4	2.0%	1.00	0.4	1.9%	1.00	0.2	1.1%	0.90	0.7	4.0%
Knee right	Sagittal	*73.3 (3.2)*	1.00	0.2	0.2%	1.00	0.2	0.3%	0.92	0.9	1.3%	1.00	0.2	0.3%
	Frontal	*11.8 (1.9)*	0.68	1.1	9.4%	0.72	1.0	8.7%	0.83	0.8	6.8%	0.15	1.8	**15.3%**
	Transverse	*18.9 (2.8)*	0.93	0.7	4.0%	0.91	0.8	4.4%	0.77	1.4	7.2%	0.78	1.3	6.9%
Ankle right	Sagittal	*46.3 (16.0)*	0.95	3.4	7.4%	0.97	2.9	6.2%	0.93	4.3	9.3%	1.00	1.0	2.2%
	Frontal	*18.5 (3.2)*	0.69	1.8	9.8%	0.56	2.1	**11.5%**	0.80	1.4	7.7%	0.83	1.3	7.2%
	Transverse	*21.3 (4.8)*	0.83	2.0	9.4%	0.81	2.1	9.7%	0.63	2.9	**13.6%**	0.81	2.1	9.9%
Hip left	Sagittal	*46.9(4.7)*	1.00	0.2	0.5%	1.00	0.2	0.5%	1.00	0.1	0.3%	0.90	1.2	2.6%
	Frontal	*15.0 (1.6)*	0.91	0.5	3.3%	0.90	0.5	3.2%	0.90	0.5	3.3%	0.40	1.3	8.5%
	Transverse	*17.2 (3.5)*	0.98	0.5	2.8%	1.00	0.8	4.5%	1.00	0.3	1.6%	0.90	0.9	5.2%
Knee left	Sagittal	*75.2 (9.3)*	1.00	0.5	0.7%	1.00	0.6	0.8%	0.98	1.2	1.6%	0.99	0.9	1.2%
	Frontal	*10.4 (2.8)*	0.60	1.8	**17.1%**	0.70	1.5	**14.9%**	0.86	1.0	**10.0%**	0.84	1.1	**10.9%**
	Transverse	*19.7 (4.5)*	0.99	0.4	2.1%	0.99	0.4	2.2%	0.97	0.7	3.8%	0.95	1.0	5.3%
Ankle left	Sagittal	*41.0 (3.7)*	0.92	1.0	2.5%	0.98	0.6	1.4%	0.96	0.7	1.8%	0.98	0.5	1.2%
	Frontal	*17.4 (4.5)*	0.87	1.6	9.1%	0.86	1.7	9.6%	0.70	2.4	**14.0%**	0.95	1.0	5.9%
	Transverse	*19.8 (3.6)*	0.68	2.0	**10.3%**	0.83	1.5	7.5%	0.75	1.8	9.2%	0.88	1.3	6.3%
Mean Sagittal		49.4 (5.9)	0.98	0.8	1.8%	0.99	0.7	1.4%	0.97	1.1	2.2%	0.96	0.7	1.7%
Mean Frontal		13.4 (2.4)	0.79	1.1	8.0%	0.80	1.1	7.8%	0.86	1.0	6.7%	0.63	1.2	9.0%
Mean Transverse		19.0 (3.7)	0.91	0.9	4.4%	0.93	0.8	4.3%	0.87	1.0	5.2%	0.90	1.1	5.4%
Mean Pelvis		12.5 (2.4)	0.99	0.1	0.8%	0.99	0.1	0.7%	1.00	0.0	0.4%	0.91	0.2	2.8%
Mean Hip		26.9 (2.8)	0.94	0.5	2.5%	0.95	0.5	2.6%	0.96	0.3	1.8%	0.76	1.1	5.2%
Mean Knee		34.8 (4.1)	0.86	0.8	5.6%	0.89	0.8	5.2%	0.89	1.0	5.1%	0.78	1.1	6.6%
Mean Ankle		27.4 (5.9)	0.82	2.0	8.1%	0.84	1.8	7.7%	0.80	2.3	9.3%	0.91	1.2	5.5%

Bold values indicate SEM% > 10%, ROM: range of motion, SD: standard deviation, ICC: intraclass correlation coefficient, SEM: standard error of measurement, SEM%: standard error of measurement in proportion to the mean.

**Table 4:** Accuracy obtained for each joint, in the 3D, in the four test movements: walking, stair ascent, stair descent, and obstacle crossing.

		Mean ROM (°)				Mean RMSE (°)				Mean ∆ROM (°)			
		Walking	Stair Ascent	Stair Descent	Obstacle Crossing	Walking	Stair Ascent	Stair Descent	Obstacle Crossing	Walking	Stair Ascent	Stair Descent	Obstacle Crossing
	Pelvis Sagittal	12.8	18.4	24.4	18.9	0.9	1.2	1.7 *	1.4	0.5	0.8	1.3	1.3
	Pelvis Frontal	6.8	13.7	20.0	13.3	0.9	2.2*	2.6 *	1.3	0.5	0.7	1.9 *	1.0
	Pelvis Transverse	17.9	20.0	47.6	27.1	1.4	1.5	3.5 *	2.0	0.2	0.6	0.8 *	0.6
Front	Hip Sagittal	50.4	83.5	69.7	104.5	2.1	2.8	7.8 *	7.3 *	1.2	1.0	2.6 *	1.5
	Hip Frontal	14.0	18.1	30.8	17.4	2.4	4.3 *	4.9 *	3.5 *	2.1	3.8	3.1	2.9
	Hip Transverse	18.4	20.6	30.0	21.4	2.2	3.1	4.2 *	4.5 *	2.0	2.2	3.0	2.8
	Knee Sagittal	73.3	98.7	101.6	119.2	3.6	5.3	12.8 *	10.2 *	1.3	4.3	2.3	5.3 *
	Knee Frontal	11.8	13.6	15.3	14.4	2.7	4.2	4.2	4.4	2.5	4.7	3.0	6.5 *
	Knee Transverse	18.9	19.1	25.3	27.8	2.4	3.8	5.4 *	5.1 *	1.9	4.2	4.0	5.6 *
	Ankle Sagittal	46.3	45.4	76.5	48.4	2.1	5.7 *	7.9 *	4.3 *	1.6	5.4	4.6	6.8
	Ankle Frontal	18.5	21.6	25.1	21.9	2.5	2.4	4.0 *	3.3	2.7	2.3	3.8	3.0
	Ankle Transverse	21.3	18.4	24.5	21.2	2.4	2.3	3.1	3.6 *	4.1	4.4	1.9	3.0
Back	Hip Sagittal	46.9	83.2	65.6	65.9	2.3	3.5	5.9 *	5.1 *	0.9	1.1	2.1	1.1
	Hip Frontal	15.0	20.2	29.9	19.6	1.8	5.2 *	4.0 *	3.3 *	1.5	4.8 *	1.9	3.6
	Hip Transverse	17.2	23.7	29.8	22.0	2.0	3.2 *	4.7 *	3.1 *	1.7	2.5	2.7	1.4
	Knee Sagittal	75.2	94.1	103.5	129.6	3.2	5.3	11.3 *	12.3 *	1.0	3.6	2.6	5.1 *
	Knee Frontal	10.4	16.2	16.3	15.3	2.7	3.7	4.4 *	4.0	2.7	2.8	4.0	4.9
	Knee Transverse	19.7	19.8	24.0	27.5	2.3	4.1 *	5.3 *	4.9 *	1.3	2.9	4.1 *	3.3
	Ankle Sagittal	41.0	50.8	70.8	43.9	2.9	4.5	9.0 *	7.2 *	1.0	6.4 *	3.5	4.6
	Ankle Frontal	17.4	18.4	22.1	22.7	2.2	2.3	3.5 *	3.9 *	2.4	2.2	3.2	2.8
	Ankle Transverse	19.8	17.8	22.1	31.8	1.9	2.3	3.9 *	5.0 *	1.8	3.6	2.2	10.5 *

ROM: range of motion, RMSE: root mean square error, ∆ROM: absolute difference in range of motion, *: significant difference in the post hoc test against the walking task.
